# Bladder triangle amyloidosis: A case report and literature review

**DOI:** 10.1097/MD.0000000000032179

**Published:** 2022-12-09

**Authors:** Zhi-Yu Yu, Lin Yan, Hua-Kang Wang, Gai Hang, Yu-Yang Wang, Quan Wen, Bo Chen

**Affiliations:** a Inner Mongolia Medical University, Huhehot, China; b Dalian Medical University, Dalian, China; c Division of Urology, Department of Surgery, Tongliao Hospital, Inner Mongolia Autonomous Region, Tongliao, China.

**Keywords:** amyloidosis, bladder tumor, case report, hematuria, pathology, urinary bladder

## Abstract

Amyloidosis is a group of benign lesions characterized by extracellular deposition of amyloid proteins. Amyloidosis lesions can occur in various organs of the body, but rarely in the urinary system. Amyloidosis in the bladder trigone is extremely rare.

**Patient concerns:**

An 80-year-old female patient presented with painless whole-course gross hematuria with reddish urine and no blood clots, accompanied by right lumbar discomfort.

**Diagnosis:**

Based on the patient’s medical history and cystoscopy findings, the relevant literature was reviewed and a preoperative diagnosis of bladder tumor was made, although bladder amyloidosis was not excluded. Postoperative pathology ultimately revealed bladder amyloidosis.

**Interventions:**

The patient underwent resection of bladder tumor and ureteral stent implantation. Postoperatively, the patient was maintained on antibiotics and oral colchicine treatment.

**Outcomes:**

Two months after surgery the patient reported that the gross hematuria had disappeared, and that the right lumbar discomfort was significantly relieved.

Cystoscopy showed no obvious recurrence in the operative area, but magnetic resonance imaging (MRI) suggested recurrence. The patient refused partial cystectomy, and the ureteral stent was removed.

**Lesson:**

The clinical manifestations of bladder amyloidosis are nonspecific, and under cystoscopy can be easily confused with bladder tumors. Accurate diagnosis of bladder amyloidosis relies on histopathology. Transurethral resection of bladder tumors or partial cystectomy is an option for surgical treatment; the latter should be performed if the ureteral opening is involved.

## 1. Introduction

Amyloidosis is a heterogeneous group of lesions characterized by extracellular deposition of amyloid proteins.^[1]^ Amyloidosis lesions can occur in various organs of the body, but rarely in the urinary system. Clinical, imaging, and cystoscopy findings associated with amyloidosis of the bladder can easily be misdiagnosed as tumors, and amyloid proteins can easily be misdiagnosed as collagen fibers. Transurethral resection of bladder tumors or partial cystectomy are surgical treatment options. The clinical and pathological data of a patient with bladder amyloidosis were retrospectively analyzed, and related diseases were discussed in conjunction with previous literature.

## 2. Case presentation

An 80-year-old woman spontaneously presented with painless gross hematuria and right lumbar discomfort the day before onset. The patient’s urine was pink with intermittent hematuria and without blood clots. No frequent urination, urgency, dysuria, pyuria, chills, and fever were observed. The patient had a history of atrial fibrillation 17 years ago; interventional therapy for liver cancer in our hospital; and intraocular lens implantation for binocular cataract 1 year prior to presentation. The patient had no family history.

Physical examination revealed no tenderness at the bilateral rib angles and no pain in the renal regions bilaterally. There was no bilateral tenderness along the ureteral course. No bladder tenderness, rebound tenderness, or dull percussions were noted in the bladder region.

Routine blood analysis showed a white blood cell count (WBC) of 3.06 × 10^9^/L and a neutrophil percentage (NEUTR) of 72.20%. Routine urine analysis showed white blood cells (LEU) +, nitrite (NIT)-positive, urobilinogen (UBG) +, and red blood cells per high power field (RBC/HPF) of 1106.50/HPF.

**Figure 1.**
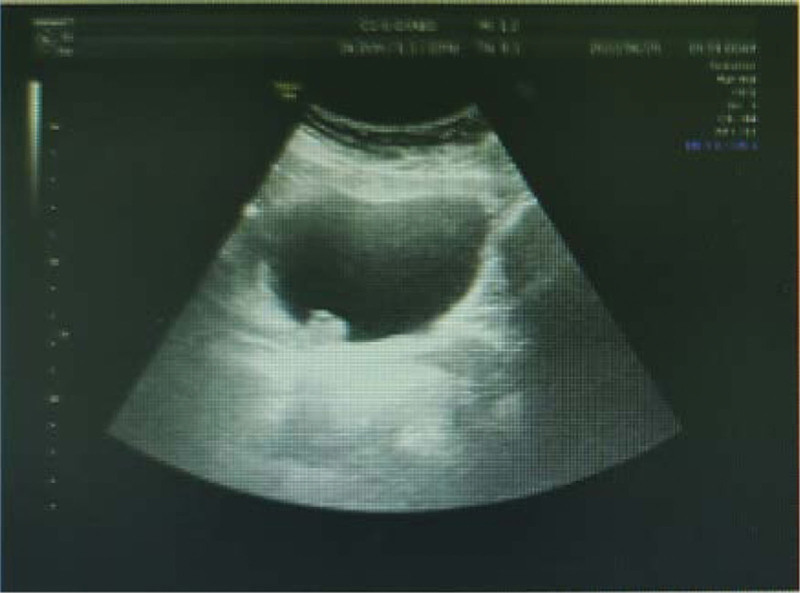
The urinary tract ultrasound. A strong echo of 2.28 × 1.99 cm in size was observed at the right ureteral entrance. The strong echo had clear boundary, irregular shape, no obvious blood flow signal, and no obvious movement even when the position was changed.

**Figure 2.**
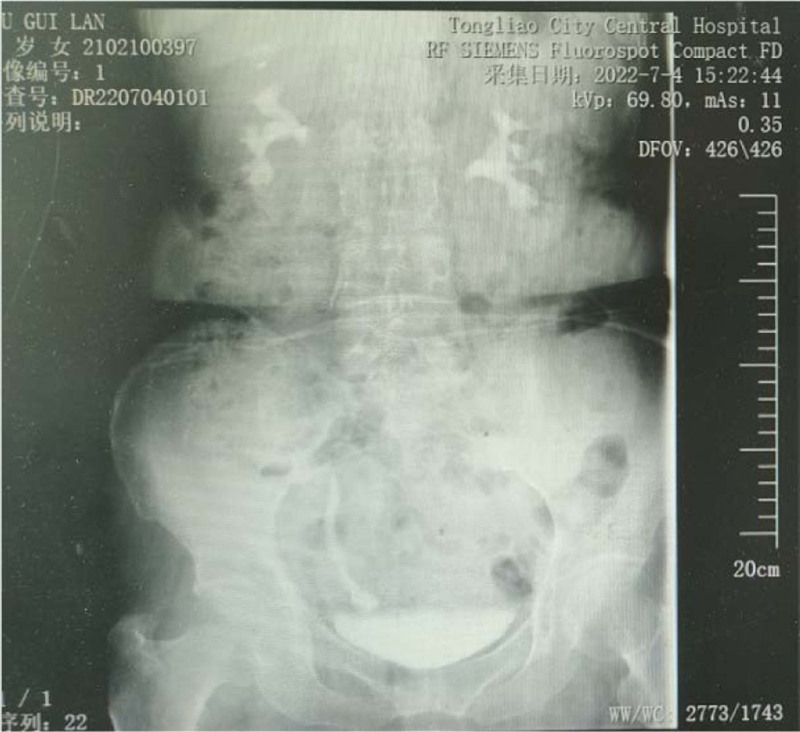
Intravenous pyelography. The middle and lower right ureter is dilated, the ureter near the bladder is spherically dilated, and the ureteral inlet is narrowed.

**Figure 3.**
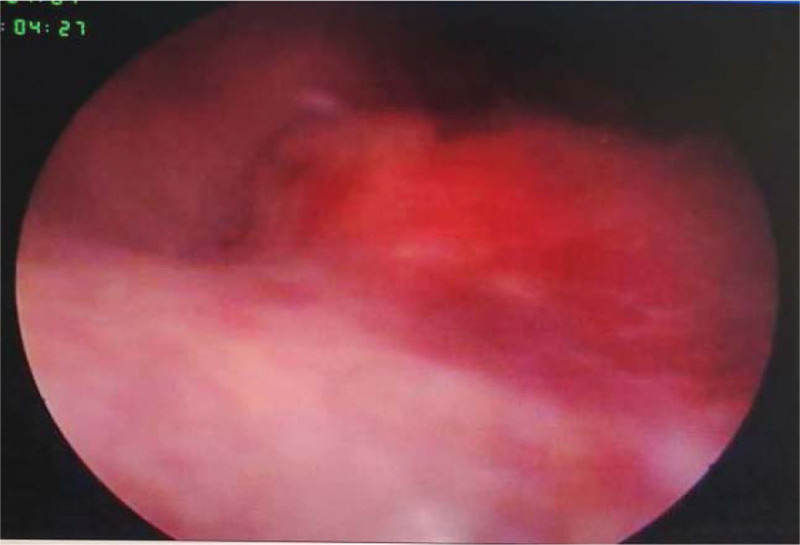
Intraoperative cystoscopy. The reddish mass protruded and looked like a volcano.

The urinary tract ultrasound report showed right renal pelvis dilatation, and the right ureter-bladder entrance was detected with a size of about 2.28 × 1.99 cm on strong echo, with clear boundaries, irregular shape, no obvious blood flow signal, and no obvious movement even with change of position. (Fig. 1) Abdominal computed tomography (CT) revealed dilation of the right renal pelvis, normal size and shape of the left kidney, no obvious abnormalities, and no abnormalities on bladder CT. Intravenous pyelography showed good renal excretion function, thickening of the middle and lower right ureters, spherical dilatation near the bladder, and an uneven superior bladder wall. (Fig. 2) Cystoscopy revealed a dark red mass approximately 2 × 2 cm in size near the right ureteral orifice, with a wide base and associated bleeding. No obvious right ureteral orifice was observed. (Fig. 3)

**Figure 4.**
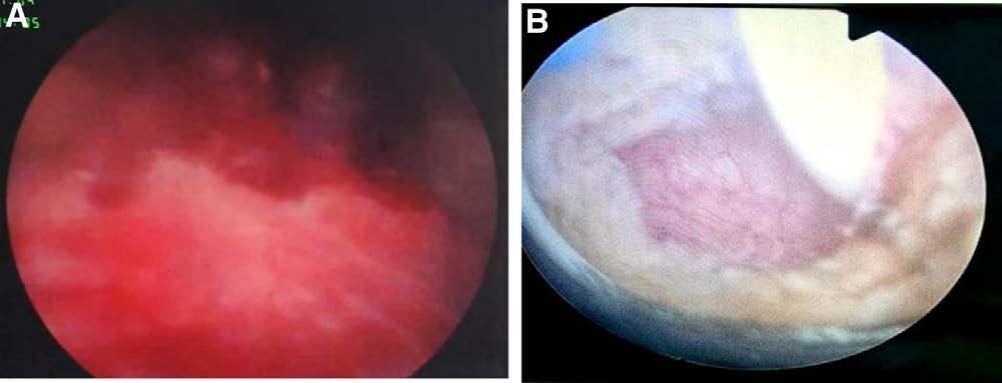
Intraoperative findings. (A) The mass completely covered the right ureteral opening with uneven surface. (B) The mass was removed, the right ureteral opening was seen, and a ureteral stent was placed.

The patient refused partial cystectomy for their own reasons and received transurethral resection of bladder tumor. Intraoperatively (Fig. 4A), the right ureteral orifice was not visible, but a dark red mass of approximately 2 × 2 cm in size was observed on the right side of the bladder, with extensive base and associated bleeding. The tumor was dissected from the walls of the bladder and the incision was extended to the muscle layer (base). After the tumor was resected a fissure was observed, and a ureteral stent was placed to determine the right ureteral orifice. The tumor was resected along the ureteral stent (Fig. 4B), and pathological examination was performed on its body and basal part. A 22F three-chamber catheter was retained postoperatively.

**Figure 5.**
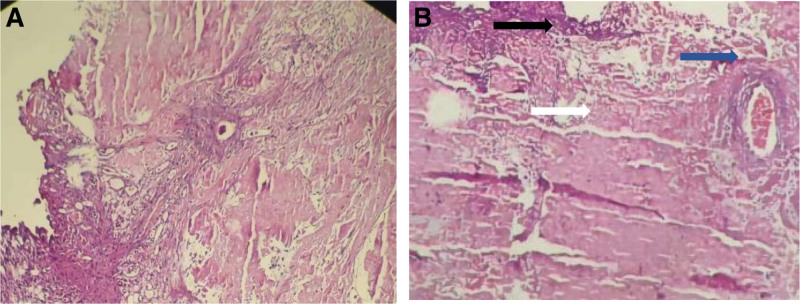
Microscopic findings (Hematoxylin and eosin staining method). (A) Urothelium, blood vessels, and amyloid are present under the same microscope (Magnification × 100). (B) Black arrows indicate urothelium, blue arrows indicate blood vessels, and white arrows indicate pink amyloid material (Magnification × 200).

**Figure 6.**
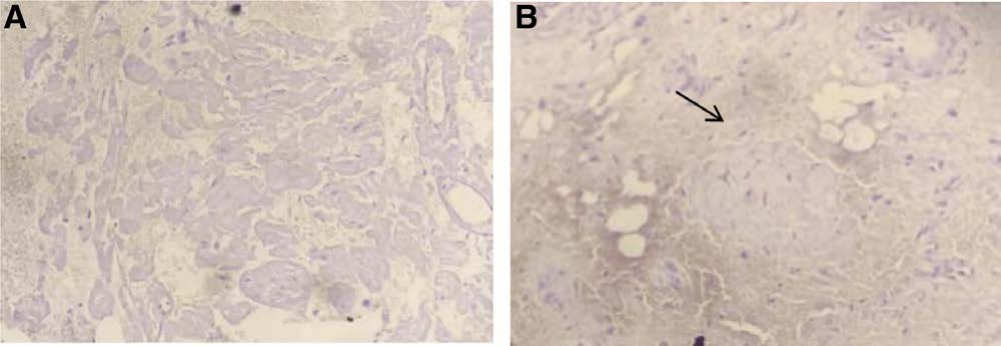
Microscopic findings (Methyl violet staining). (A) The methylviolet stain was positive, and the amyloid material was purplish blue (Magnification × 200). (B) Purple-blue rose-like amyloid protein shown by arrow (Magnification × 200).

**Figure 7.**
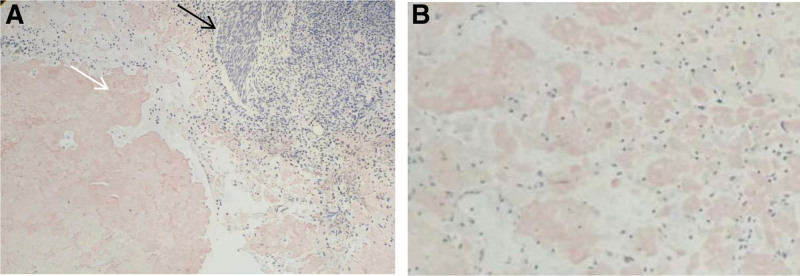
Microscopic findings (Congo red staining under white light). (A) The black arrow indicates the urothelium, and the white arrow indicates amyloid (Magnification × 100). (B) Amyloid protein is brick red (Magnification × 200).

**Figure 8.**
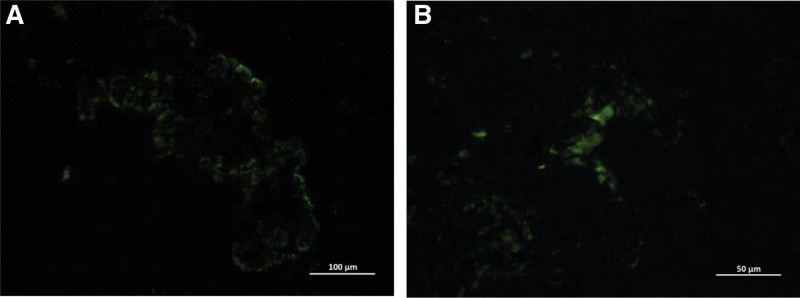
Microscopic findings (Congo red staining under polarized light). (A) Amyloid protein presenting apple green birefringent light. (B) Amyloid protein presenting apple green birefringent light.

The pathological report and hematoxylin-eosin staining (Fig. 5) suggested acute and chronic bladder inflammation, interstitial inflammatory cell infiltration, and no visualization of structural material using the powder dye, indicating amyloidosis. Methyl violet staining was positive (Fig. 6), and the amyloid protein deposit was a purple-red rose-like substance. Congo red staining showed brick-red amyloid protein deposits under white light (Fig. 7), and yellow-green birefringence associated with the amyloid proteins was observed under polarized light (Fig. 8).

**Figure 9.**
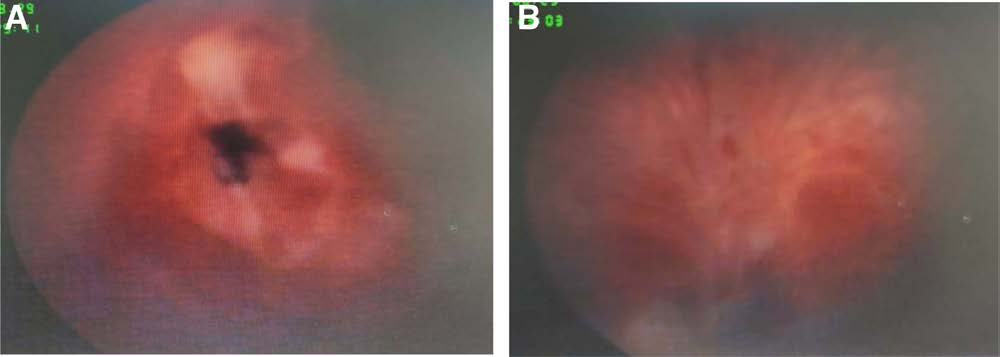
Postoperative cystoscopy review. (A) The right ureteral opening was clearly visible, and scar tissue hyperplasia was seen around the right ureteral opening. (B) Scar in the electrotomy area.

**Figure 10.**
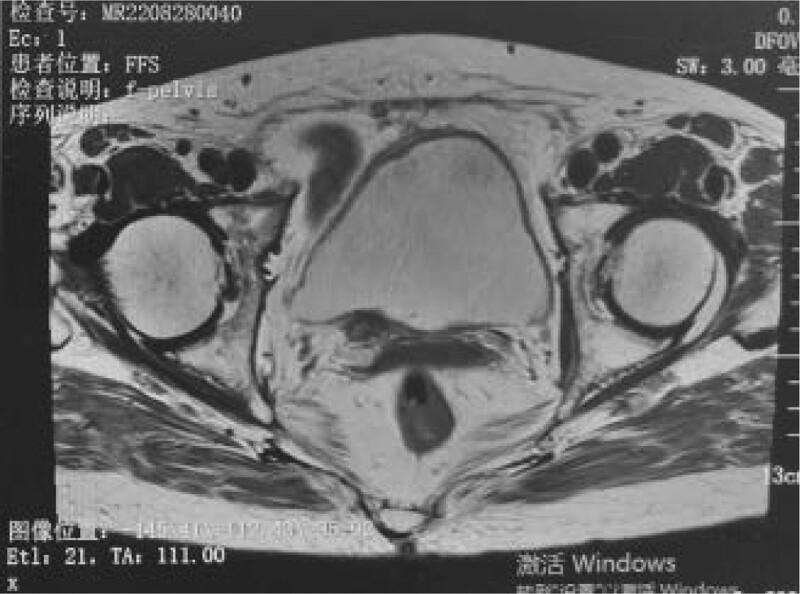
Postoperative MRI review. Limited thickening of the bladder wall at the entrance of the right ureter, low T2-weighted signal in the lesion area, and the possibility of recurrence. MRI = magnetic resonance imaging.

Postoperatively, the patient was maintained on antibiotics, oral colchicine treatment, and indwelling catheterization for 7 days. The patient was discharged after recovery, and oral colchicine treatment was continued after discharge. Two months after surgery, the patient reported that the gross hematuria had disappeared and that the symptoms of lumbar discomfort were significantly relieved. Although follow up cystoscopy showed no recurrence (Fig. 9), magnetic resonance examination revealed localized thickening of the right ureteral entrance, suggesting recurrence (Fig. 10). However, the patient refused to undergo partial cystectomy, and the ureteral stent was removed.

## 3. Discussion

Amyloidosis was first described by Rokitansky in 1853,^[2]^ as a general term referring to a disease characterized by abnormal deposition of various amyloid proteins in the extracellular space of body organs or tissues. A common feature of this group of diseases is that a uniform microfibrillar substance is deposited in tissues and organs, causing abnormal organ structure and function and corresponding clinical symptoms and signs. Amyloidosis is divided into 2 categories: systemic and localized, according to the affected organs or tissues.^[3]^ Systemic amyloidosis involves multiple organs of the body, including the gastrointestinal tract, cardiovascular system, striated muscles, and adipose tissue. Many studies have suggested that it is related to chronic monoclonal inflammatory responses, or abnormal immune function.^[4]^ Localized amyloidosis is more common in the lungs, throat, tongue, and reproductive organs.^[5]^

Primary localized urinary tract amyloidosis is rare in the clinical setting. Since urinary amyloidosis was first reported in 1897, fewer than 200 cases have been reported worldwide.^[6]^ These lesions can involve the kidneys, ureter, bladder, and urethra. In 1965, Li Yantang et al^[7]^ first reported primary localized bladder amyloidosis in China; since then, there have been fewer than 100 similar reports. Bladder amyloidosis is more common in adults. Caldamone^[8]^ collected data of 46 patients and found that the onset age was between 28 and 80 years, with an average onset age of 53 years. Researchers have reported that the peak age of onset is between 50 and 60 years, with a male to female ratio of 3:1.^[9]^ The etiology of bladder amyloidosis remains unclear. Currently, most researchers believe that it is caused by a chronic inflammatory response and abnormal immune function. Other scholars believe that it may be a long-term chronic infection of the urinary tract, or repeated inflammation of the mucosa and submucosa that causes a large number of local lymphoid plasma cells to accumulate and proliferate in a monoclonal form, thereby producing immunoglobulin light chains, and forming the amyloid proteins deposited in the tissue.^[10,11]^ In the reported patient’s urinalysis, we noted a large number of white blood cells and nitrite (NIT) positivity, as well as inflammatory cell infiltration in her pathological specimens.

The study found that >80% of the patients with primary localized bladder amyloidosis had painless gross hematuria as the initial symptom, similar to bladder tumors. This group of patients was also hospitalized with hematuria as their primary complaint. As cystoscopy results resemble those of a bladder tumor, bladder amyloidosis is easily misdiagnosed in clinical practice, and only biopsy or postoperative pathology can confirm its diagnosis.^[12,13]^ This was also observed during the cystoscopy of the reported case, as there were no significant differences between what was visualized on cystoscopy in this patient and bladder tumors. Therefore, the patient was diagnosed with a bladder tumor before the surgery, and amyloidosis was only confirmed after pathological examination and special stainings. Because there was no systemic disease, no other predisposing factors, and no family history, the patient was diagnosed with primary localized lower urinary tract amyloidosis. Studies have reported that cystoscopy can detect multiple localized, slightly yellow polypoid disease, mucosal spotted hemorrhage, ulcer formation,^[14]^ and papillary lesions.^[15]^ However, these were not observed in the present case.

The distribution of amyloidosis in the bladder can involve all the bladder walls. According to statistics, 68% of the bladder anterior wall, 26% of the triangular area, and 6% of the other parts are involved.^[16]^ The lesion in this patient involved the bladder trigone and invaded the ureteral orifice, which has not been reported previously. No positive findings were found on urine exfoliative cytology. In few cases, amyloid proteins with no fixed shape appeared around exfoliated cells.^[17]^ Color Doppler ultrasound, intravenous urography, and CT cannot effectively distinguish the disease from bladder tumors. We had the same experience during our patient’s treatment. It has been reported that magnetic resonance imaging (MRI) of amyloidosis lesions shows low signal intensity on T2-weighted images, while bladder cancer shows moderate to strong signals.^[18]^ This may be related to the active proliferation of malignant tumors, high cell content in tissues, large cell volume, tight arrangement, enlargement of the nucleus, increased nuclear-to-cytoplasmic ratio, and limited water diffusion. In this case, the lesion also showed a low signal on the T2-weighted image after MRI examination. Therefore, we believe that preoperative bladder MRI may be the ideal imaging modality for this disease.

Amyloid substances that cause amyloidosis are mainly deposited in the urinary tract mucosa, submucosa, and superficial muscle layer, protruding outward, and growing in a tumor-like appearance. Amyloidosis is mainly diagnosed based on histopathological examination and special stainings, including Congo red, methyl violet, and potassium permanganate-Congo red staining. Histological examination revealed amorphous eosinophilic protein depositions.^[19]^ The pathological features were mainly observed by light microscopy. Routine hematoxylin-eosin staining showed that the thickness of the urinary tract epithelium was different, and eosinophilic amyloid deposits were observed in the lamina propria and muscular layers. Eosinophils, lymphoid plasma cells, and giant cell infiltration were observed in the lesion area, and the Congo red staining was brick-red. Polarized light was used to observe the yellow-green birefringence of the amyloid deposits, which was consistent with our pathological results. Therefore, primary amyloidosis was confirmed.

Because the etiology of primary amyloidosis is still unknown, there is a lack of effective treatments; therefore, surgery is the primary choice of treatment. For smaller lesions, transurethral resection, transurethral electrocautery, or laser treatment is the first choice. Partial cystectomy or total cystectomy can be performed in cases with large lesions and severe hematuria. The patient refused partial cystectomy because of advanced liver cancer and underwent transurethral resection of the bladder tumor. Drug therapy is often used to prevent recurrence after surgery. Commonly used drugs include oral colchicine or cepharanthine, and bladder perfusion with dimethyl sulfoxide.^[20]^ Dimethyl sulfoxide is an industrial solvent. In 1976, Isobe and Osserman demonstrated its ability to degrade amyloid fibrils in vitro. Since then, oral medications and intravenous injections have been used to treat primary and secondary amyloidosis, improving clinical symptoms. The patients did not experience any other side effects except for a garlic-like taste. Recently, some researchers have successfully used dimethyl maple to treat such patients.^[21]^ Its specific usage included an initial injection of 40 mL of lidocaine into the bladder, emptying the bladder 30 minutes later, followed by perfusion of 50 mL of 50% DMSO into the bladder and retaining it for 30 minutes. The procedure was repeated once every 2 weeks, 3 months is a course of treatment, generally from 6 months to 1 year. Some researchers have also applied 10% dimethyl sulfoxide bladder perfusion solution achieving the same effects.^[22]^ The author of the current study failed to find medical dimethyl sulfoxide in China; therefore, the patient was only prescribed with oral colchicine (2 mg/d) to prevent recurrence after surgery. Two months after surgery the patient reported that the gross hematuria had disappeared, and that the symptoms of lumbar discomfort were significantly relieved. Although follow up cystoscopy showed no evidence of recurrence, the MRI findings were suggestive of recurrence. Thus, bladder amyloidosis is a highly recurrent disease. The pure bladder tumor electricity cut method can solve this problem to some extent, but the forward still has a tendency to relapse. Especially for bladder amyloidosis involving ureteral opening, partial cystectomy should be performed at the beginning to completely solve the primary lesion, and in severe cases, total cystectomy should be performed to avoid the loss of follow-up after electrotomy, resulting in undetected hydronephrosis and affecting renal function.

In conclusion, bladder amyloidosis is a rare benign bladder lesion with nonspecific clinical manifestations that under cystoscopy can be easily confused with bladder tumors, and its misdiagnosis rate is high. The diagnosis of bladder amyloidosis depends on histopathological examination. This disease is prone to recurrence. Transurethral cystectomy or partial cystectomy are surgical treatment options, and partial cystectomy should be performed if the ureteral opening is involved. Regular intravesical infusion of dimethyl maple or oral colchicine should be performed after surgery, and regular follow-up is crucial.

## Author contributions

**Conceptualization:** Bo Chen.

**Data curation:** Gai Hang.

**Software:** Lin Yan.

**Validation:** Yu-Yang Wang.

**Visualization:** Quan Wen.

**Writing—original draft:** Hua-Kang Wang.

**Writing—review & editing:** Zhi-Yu Yu.
